# The pluripotency marker Rex-1/Zfp42 negatively regulates expression of endogenous retroviruses (ERV) and ERV-associated genes

**DOI:** 10.1186/1742-4690-8-S2-P61

**Published:** 2011-10-03

**Authors:** Diana Guallar, María Climent, Raquel Pérez-Palacios, Ignacio García-Tuñón, P Muniesa, Jon Schoorlemmer

**Affiliations:** 1 Facultad de Veterinaria, Universidad de Zaragoza, Instituto Aragonés de Ciencias de la Salud (Aragon Health Sciences Institute), Spain; 2 Departamento de Anatomía y Embriología, Facultad de Veterinaria, Universidad de Zaragoza, Spain; 3 Programa Aragonés de Medicina Regenerativa (PAMER), Instituto Aragonés de Ciencias de la Salud, Spain

## Background

Expression of the Yyl-related zinc finger protein Rex1 is restricted to primary spermatocytes, the preimplantation embryo and various types of pluripotent cells in culture. In line with its association to more pluripotent subpopulations, it is widely used as a marker for embryonic stem cells. Rex1 has been reported to regulate Tsix expression in ES cells, and imprinted genes during preimplantation development. Almost half of the mammalian genome is derived from retroelements, of which 25% are endogenous retroviruses (ERVs). During preimplantation development, these elements are silenced by epigenetic modifications, but the factors controlling these events are largely unknown.

## Materials and methods

We have studied the role of Rex-1 in mouse ES cells and during mouse preimplantation development, using microinyection of either over-expressing plasmids or siRNAs. Gene expression was measured by (semi)-quantitative PCR. To identify additional genes regulated by Rex-1, we have carried out genome-wide chromatin association studies in mouse ES cells. Gene association was subsequently analyzed by chromatin-ímmunoprecipitation assays.

## Results

In ESC with reduced expression of Rex-1 we did not observe changes in the expression of a selection of pluripotency and differentiation markers, in contrast to increased expression of endogenous retroviruses muERV-L and MusD. We probed for Rex-1 binding to these elements using ChIP assays in ES cells. Rex-1 strongly associates to MuERV-L and to a lower extent to IAP and MusD elements. Furthermore, association was not seen in Rex-1 depleted or RA-treated ES cells. Rex-1 also regulates muERV-L expression in vivo, as we show altered levels upon transient gain-and-loss of Rex-1 function in preimplantation embryos. Preliminary analysis of the genomic loci identified indicates that Rex-1 associates with a set of genes regulated by degenerated viarl LTRs, whose expression is highly regulated during preimplantation development, generally displaying peak expression levels in the blastocyst. Surprisingly these same genes are not necessarily highly expressed in mouse ES cells, although Rex-1 depletion does alter expression levels.

## Conclusions

Our results suggest that Rex-1 regulates expression of ERV and ERV LTR-associated genes in mouse ES cells and during mouse preimplantation development. We hypothesize that Rex-1 has evolved as a regulator of endogenous retroviral transcription. We propose that the Yy1 family of transcription factors and Rex-1 in particular may have co-evolved with ERV. Such a model suggests that Yy1 family members may be instrumental to control the spread of silencing from repetitive elements to neighbouring genes, or to orchestrate ERV-mediated control of cellular genes during preimplantation development and desease. We provide a potential explanation for the strong conservation of Yy1 family members, and a potential co-evolution mechanism that has allowed co-optation of ERV-derived cis elements for specific developmental processes.

